# Epidemiological, Pathophysiological, and Clinical Considerations on the Interplay between Thyroid Disorders and Type 2 Diabetes Mellitus

**DOI:** 10.3390/medicina59112013

**Published:** 2023-11-16

**Authors:** Gregory Grigoriadis, Theocharis Koufakis, Kalliopi Kotsa

**Affiliations:** 1 Medical School, Aristotle University of Thessaloniki, 54124 Thessaloniki, Greece; gregorygrigoriades@gmail.com; 2 Second Propaedeutic Department of Internal Medicine, Hippokration General Hospital, Aristotle University of Thessaloniki, 54642 Thessaloniki, Greece; 3 Division of Endocrinology and Metabolism and Diabetes Center, First Department of Internal Medicine, Medical School, Aristotle University of Thessaloniki, AHEPA University Hospital, 54636 Thessaloniki, Greece; kkalli@auth.gr

**Keywords:** type 2 diabetes mellitus, insulin resistance, thyroid dysfunction, hyperthyroidism, hypothyroidism, thyroid cancer

## Abstract

Thyroid disorders (TD) and diabetes mellitus (DM) are the two endocrinopathies with the highest prevalence in the general population that frequently coexist. Thyroid dysfunction is more common in people with type 2 diabetes mellitus (T2DM) compared to normoglycemic individuals. Untreated TD can impair glycemic control, increasing the risk of diabetes complications. Hyperinsulinemia can affect the morphology of the thyroid gland by promoting the proliferation of thyroid tissue and increasing the size of thyroid nodules. Metformin can confer benefits in both endocrinopathies, while other antidiabetics, such as sulfonylureas, can negatively affect thyroid function. Animal and human observational data suggest an increased risk of medullary thyroid carcinoma after treatment with glucagon-like peptide-1 receptor agonists. However, randomized trials have so far been reassuring. Furthermore, some observational studies suggest an association between thyroid cancer and T2DM, especially in women. This narrative review aims to shed light on the epidemiological, pathophysiological, and clinical aspects of the interplay between TD and T2DM. Taking into account the important clinical implications of the coexistence of T2DM and TD, proper screening and management strategies are needed for both endocrinopathies to ensure optimal patient care.

## 1. Introduction

Diabetes mellitus (DM) is the most common endocrinopathy worldwide and is characterized by insulin resistance and/or deficiency, leading to hyperglycemia. According to the International Diabetes Federation, in 2021, the global prevalence of DM in the adult population was approximately 10.5% (537 million), and this number is expected to increase to 643 million by 2030 [1]. Regarding the prevalence of thyroid disorders (TD), a large meta-analysis estimated that they affect 3.82% of the general population [2]. Other studies estimate that the prevalence of TD in Europe and the United States is ~6.6% in adults [3,4], increases with age, and is higher in women than in men.

The coexistence of DM and TD is frequently observed in daily practice. A higher prevalence of TD has been documented in people with DM compared to normoglycemic individuals, while patients with both endocrinopathies have poorer glycemic control and are more vulnerable to the development of complications [5]. There are several studies that verify the link between type 1 diabetes mellitus and autoimmune TD, thus demonstrating common pathogenetic mechanisms between the two entities [6]. However, there is a paucity of data on the complicated underlying mechanisms that link T2DM and TD [7]. This narrative review aims to shed light on the epidemiological, pathophysiological, and clinical aspects of the interplay between TD and T2DM, according to the latest evidence.

## 2. Epidemiological Data on the Coexistence of Type 2 Diabetes Mellitus and Thyroid Disorders

The prevalence of TD is significantly higher among patients with T2DM compared to the general population, ranging from 9.9 to 48% in various studies [8,9]. In a study that included 1310 adults with T2DM attending a diabetic outpatient clinic, Perros et al. estimated that the prevalence of TD was 13.4% and was higher compared to that reported in the general population. TD was identified by estimating serum-free thyroxine (FT4) and thyroid-stimulating hormone (TSH) concentrations [10]. A study in Jordan estimated the prevalence of TD at 12.5% in patients with T2DM. In this study, 908 patients with T2DM and 304 normoglycemic controls were recruited, and thyroid function was evaluated by measurements of FT4, free triiodothyronine (FT3), and TSH. Furthermore, in 600 patients with T2DM and 282 controls, thyroid autoantibodies were evaluated. When comparing the T2DM group with the control group, the positivity for thyroid peroxidase antibodies (anti-TPO) was found to be 8.3% versus 10.3%, and the positivity for both anti-TPO and thyroglobulin antibodies (anti-TG) was found to be 2.5% versus 6%, respectively. Although the prevalence of thyroid dysfunction was higher in the T2DM group, thyroid autoantibodies were found to be more common in the control group [11].

Another study in Saudi Arabia revealed that the prevalence of TD was 16% in patients with T2DM. This study included 100 individuals with T2DM and 100 age- and sex-matched normoglycemic controls. Thyroid autoimmunity (anti-TPO, anti-TG) was detected in 10% of individuals with T2DM versus 5% of controls. Additionally, antibodies to glutamic acid decarboxylase 65 (GAD65ab) were found in 26% of patients with T2DM and 2% of controls. In GAD65ab-positive patients with T2DM, thyroid autoimmunity was observed in 27% versus 4% in GAD65ab-negative patients, and TD was diagnosed in 42% versus 7%, respectively [12]. A Greek study among 1092 patients with T2DM reported that TD was present in 12.3%. Women were affected more frequently than men. TD was defined as being under treatment with T4, T3, antithyroid drugs, or having a history of thyroidectomy and radioactive iodine treatment [13]. Celani et al. estimated a 31.4% prevalence of TD among 290 patients with T2DM hospitalized due to poor glycemic control or a recent diagnosis of DM. The prevalence of TD was significantly higher in women compared to men and in insulin-treated patients than in those receiving oral hypoglycemic agents [14].

Cheng et al. [15] conducted a systematic review of the correlation between subclinical hypothyroidism (SCH) and T2DM and estimated the prevalence of SCH at 10.2% in individuals with T2DM. Additionally, SCH was found to be more prevalent in individuals with T2DM compared to the general population, where the prevalence of SCH is 4–9% [9,16,17]. These findings are consistent with a study by Tamez-Perez et al. that showed a prevalence of 5.7% of overt hypothyroidism in patients with T2DM, compared to a prevalence of 1.8% in normoglycemic individuals, highlighting a strong correlation between T2DM and hypothyroidism [18]. In the T2DM population, individuals over 60 years of age and women had an increased risk of SCH. Table 1 provides an overview of studies reporting on the prevalence of coexistence of T2DM and TD.

## 3. The Interplay between Thyroid Hormones and Glucose Homeostasis

Thyroid hormones (THs) affect the regulation of glucose homeostasis and lipid metabolism through both the central nervous system and directly in peripheral target organs such as the liver, skeletal muscle, pancreatic beta cells, and white and brown adipose tissues [19]. In particular, THs enhance glucose absorption by the gastrointestinal tract and increase hepatic gluconeogenesis through increased activity of the enzyme phosphoenolpyruvate carboxykinase (PEPCK) [20]. The consequences of increased glycogenolysis and increased hepatic glucose output are hyperinsulinemia and glucose intolerance, leading to insulin resistance [21]. Furthermore, THs increase lipolysis in adipose tissue, leading to a slight elevation in serum-free fatty acid levels within the normal range. Excess THs can even cause insulin resistance [20]. THs increase glucose uptake by skeletal muscle through expression of the GLUT4 gene and increase insulin and glucagon secretion by beta and alpha pancreatic cells, respectively [20].

A large-scale cross-sectional study by Gu et al. [7] demonstrated that TSH, FT4, and FT3 levels correlate with the risk of T2DM, even if they are within the normal reference range. More specifically, after adjustment for various confounders in both men and women, there was a higher prevalence of T2DM among the adult population in individuals with reduced FT3 levels, FT3/FT4 ratio, and increased levels of FT4, and this finding was independent of age. Furthermore, a significant inverse correlation was observed between TSH and the prevalence of T2DM in men, although this observation was not replicated in women.

TSH stimulates deiodinase expression and activity [22,23]. Elevated peripheral deiodinase activity increases the conversion of FT4 to FT3 and the basal metabolic rate, which is important for the regulation of adipose tissue homeostasis [24,25]. Conversely, a suppression of peripheral deiodinase activity lowers the basal metabolic rate. Therefore, the FT3/FT4 ratio can be considered as a marker of peripheral deiodinase activity in T2DM.

Insulin resistance and excess insulin release observed in DM induce the proliferation of the thyroid gland, thus increasing the incidence, size, and volume of thyroid nodules [26,27]. Furthermore, among those with T2DM and thyroid nodules, females have a larger nodule volume and size that are positively associated with the magnitude of insulin resistance [27]. Periodic changes in female endocrine hormones may be related to the higher frequency of thyroid nodules in women [28]. In patients with T2DM, insulin resistance is a risk factor for thyroid nodules. Reducing insulin resistance can slow their growth rate and decrease both their volume and size [27].

DM interferes with thyroid function by modifying TSH levels and inhibiting the conversion of T4 to T3 in peripheral tissues [26,29]. In individuals with DM and normal thyroid function, the nocturnal TSH peak has been found to be absent or weak, and the TSH response to thyrotropin-releasing hormone (TRH) is also impaired [30]. Adipose tissue releases several hormonal mediators, such as leptin, which have been found to be elevated in T2DM patients. Leptin stimulates the hypothalamus–pituitary–thyroid axis, which in turn raises TSH levels [31]. Moreover, acute situations such as diabetic ketoacidosis can confound laboratory tests of thyroid function by decreasing T3 and T4 levels while TSH levels remain unchanged [32]. In individuals with hypothyroidism, the efficacy of TH replacement could be affected by coexisting DM [26].

Visceral adiposity is positively correlated with insulin resistance and the risk of T2DM [33]. TSH levels have been proven to be associated with the degree of obesity in people with normal thyroid function, and TSH levels are higher in people with obesity compared to controls [34]. The main mechanism that leads to the elevation of TSH in this population is the increased secretion of leptin by adipose tissue [35]. A positive correlation has been reported between serum leptin and TSH levels [36,37,38]. Furthermore, it has been shown that there is a significant and positive association between serum TSH, even within the normal range, and BMI. Patients with SCH who lost weight after bariatric surgery improved or normalized their TH levels [39]. Therefore, it is evident that people with DM have higher levels of leptin secretion, which can stimulate TSH synthesis through the hypothalamic–pituitary–thyroid axis [40]. Excess insulin can modulate glycemic levels and, furthermore, may induce an elevation of TRH and TSH [41]. From a clinical perspective, complications such as diabetic nephropathy, diabetic retinopathy, peripheral arterial disease, and diabetic peripheral neuropathy were observed to occur more frequently in individuals with T2DM and SCH [15].

## 4. Specific Thyroid Disorders and Type 2 Diabetes Mellitus

### 4.1. Hyperthyroidism

TH excess leads to several alterations in peripheral organ targets and induces hyperglycemia and insulin resistance. THs exert a key role in hepatic glucose metabolism, with stimulative effects on liver glucose production and insulin requirement. They stimulate the hepatic expression of the glucose transporter GLUT2, resulting in increased hepatic glucose output [42]. Also, in hyperthyroid individuals, there is an increase in mRNA expression and activity of the enzyme PEPCK and other hepatic gluconeogenic enzymes, which enhance gluconeogenesis and glycogenolysis, eventually leading to the development of liver insulin resistance [43]. Additionally, epinephrine and glucagon have gluconeogenic and glycogenolytic effects on the liver, which are assisted by THs through the affection of b2-adrenergic receptor mRNA and the suppression of inhibitory G protein RNA of the adenylate cyclase cascade [44]. Furthermore, increased lipogenesis and lipolysis are induced by THs and exaggerate the liver glucose and lipid metabolism imbalance, leading to insulin resistance [42].

Unlike the liver, THs act synergistically with insulin in the peripheral tissues. T3 upregulates the expression of GLUT4, adenosine monophosphate-activated protein kinase, and acetyl coenzyme A carboxylase, which are involved in basal and insulin-stimulated glucose transport, utilization, and glycolysis in skeletal muscle [45,46]. T3 upregulates mitochondrial uncoupling protein (UCP)3, leading to the increased energy expenditure seen in hyperthyroidism [47]. Skeletal muscle and adipose tissue exert opposite effects. Skeletal muscles produce several myokines that affect adipose tissue, while adipose tissue delivers adipokines that can modulate the insulin sensitivity of skeletal muscle. Both hyperthyroidism and hypothyroidism intervene in this pathway, thus contributing to insulin resistance [48]. Insulin-stimulated glucose oxidation rate increases in muscle and adipose tissue of patients with hyperthyroidism. Increased lipolysis is also observed in the adipose tissue of hyperthyroid individuals, leading to elevated free fatty acid levels. A hypermetabolic state is seen in patients with hyperthyroidism, and pre-existing glucose intolerance can be aggravated. Hyperthyroid individuals are at increased risk of severe hyperglycemia, and in the context of insulin deficiency, elevated lipolysis, and hepatic b-oxidation may induce ketoacidosis [20].

In the normal range, T3 influences insulin production by pancreatic beta cells since neonatal beta cells have TH receptors, and their exposure to T3 promotes the activation of the transcription factor MAFA, which stimulates beta cell maturation. However, in hyperthyroid rats, the pancreas is seen to increase, but both the islets’ capacity and the overall number of insulin-positive cells are decreased. A significant decline in islet function and decreased glucose-induced insulin production appear to be caused by a decrease in beta cell mass and dysfunction of the insulin secretory pathway, which involves two essential components: ATP-sensitive K^+^ and L-type Ca^2+^ channels. Abnormal glucose tolerance is not caused by insulin resistance but rather by a defective pancreatic beta cell response to glucose [49,50,51]. Furthermore, increased insulin degradation and accelerated insulin clearance are observed in individuals with hyperthyroidism [52].

### 4.2. Hypothyroidism

Patients with hypothyroidism may also develop insulin resistance, although there are some crucial differences compared to those with hyperthyroidism. Clearly, low levels of THs affect several organs. First, there is reduced glucose absorption by the gastrointestinal tract. Decreased hepatic glucose output is observed due to diminished liver and muscle gluconeogenesis and glycogenolysis [53]. Regarding peripheral tissues, reduced insulin-stimulated glucose transport, glucose disposal, and utilization are observed in hypothyroid individuals due to insulin resistance [54]. The glucose oxidation rate and glycogen production are reduced as well. Insulin resistance, both fasting and postprandial, has been detected in patients with subclinical and overt hypothyroidism [55]. Insulin resistance may be correlated with reduced expression of the GLUT4 transporter, elevated free fatty acids, and impaired leptin action.

Regarding beta cell function, glucose-induced insulin secretion is increased, and several studies have shown that insulin levels are elevated in hypothyroidism [56]. Furthermore, the half-life of insulin is prolonged due to reduced insulin clearance by the renal system [57]. In people who have DM and hypothyroidism and are treated with insulin, an adjustment in insulin dosage might be needed. Diminished renal insulin clearance leads to higher insulin levels; therefore, exogenously administered insulin requirements might be lower [58,59].

### 4.3. Thyroid Malignancies

The full spectrum of risk factors for TC is not well established. Environmental exposure to neck irradiation, inadequate iodine intake, family history of TC, and lifestyle factors are believed to be associated with the increase in prevalence [60,61,62,63,64,65]. The role of other risk factors in the development of TC must be clarified. Therefore, a possible causable role of T2DM and obesity in TC is considered. Yeo et al. performed a systematic review and meta-analysis to investigate the association between T2DM and the incidence of TC. Data from 13 studies were extracted, showing that T2DM was correlated with a notable increase in the risk of TC by approximately 20%. Furthermore, women with T2DM experienced a 30% higher risk of TC (1.38-fold increased risk) compared to their counterparts without DM, but this finding was not replicated in men [66].

The worldwide origin of the studies provided considerable population heterogeneity and indicated that the risk associated with DM was more prominent among women in areas with a high incidence of TC compared to other geographical areas [66]. Another more recent meta-analysis by Yin et al. investigated the association between TC and insulin resistance, metabolic syndrome, and its components. In accordance with the findings of the previous study, an increased incidence of TC was observed in women but not in men. Insulin resistance, dysglycemia, high body mass index (BMI), and hypertension were shown to significantly increase the incidence of TC. More particularly, insulin resistance has the highest risk estimate among components of the metabolic syndrome and is related particularly to PTC. There was a positive correlation with TC prevalence in both women and men with BMI > 25 kg/m^2^, but this correlation was stronger in men [67]. On the contrary, a large prospective study [68] and a pooled analysis of five prospective studies [69] did not detect a significant association between DM and TC. A previous review of the literature indicated that any association between T2DM and TC is probably weak [70].

There are some suggestive molecular biological mechanisms that could explain the association between DM and the increase in the incidence of TC. First, hyperinsulinemia observed in patients with T2DM can impede cell apoptosis and promote cell proliferation through stimulation of insulin and the insulin-like growth factor-1 (IGF-1) pathway [71]. Increased insulin levels may affect TC risk, which is mediated by insulin receptors overexpressed in tumor cells and tissues [72]. In addition, insulin can play a key role in the promotion of thyroid carcinogenesis through the stimulation of mitogen-activated protein kinase (MAPK) and phosphatidylinositide 3-kinase pathways by mimicking IGF-1 and binding to the IGF-1 receptor [72]. Regarding stimulation of the IGF1 signaling pathway, hyperinsulinemia seen in T2DM reduces IGF-binding protein levels and enhances bioavailable IGF levels afterward. Therefore, a suggestive mechanism is that hyperinsulinemia directly increases cancer progression through overexpressed insulin receptors or indirectly through IGF-1 signaling [73].

Second, long-term exposure to higher TSH levels was correlated with an increased probability of DTC and a more aggressive tumor stage [74]. Elevated TSH levels are observed to be three times more frequent in people with T2DM compared to controls without DM [58]. Overproduction of THs promotes thyroid inflammation and TC through genomic and nongenomic effects. The genomic effect stimulates thyroid carcinogenesis through specific nuclear receptors; however, activation of the MAPK signaling pathway has been suggested to be involved in the pathogenesis of PTC. Furthermore, a novel pathway mediated by a membrane receptor located in integrin αVβ3 has been detected, which in part explains the proliferative and angiogenic effects of THs [75]. A different mechanism involves the impact of hyperglycemia on tumor cell growth and proliferation through increased pro-inflammatory and oxidative stress [70,76].

Glucose can increase the production of reactive oxygen species, especially nitric oxide [77]. Vitamin D deficiency is observed in 70% of individuals with DM [70] and, in this state, inactivation of deiodinase II leads to decreased glucose transporter 4 (GLUT4) transcription by skeletal muscle and adipose tissue, thus inducing insulin resistance and thyroid carcinogenesis [70,78]. In addition, in people living with obesity, adipocytokines and cytokines are secreted by adipocytes and inflammatory cells, respectively, infiltrating adipose tissue and contributing to the pathogenesis of insulin resistance. Adipocytokines are generally upregulated with increasing fat mass, in contrast with adiponectin, which is downregulated [79]. The most abundant and most investigated adipocytokines, leptin and adiponectin, may also be related to an increased incidence of TC. The increased levels of leptin that are observed in obesity are correlated with increased cancer generation. Rehem et al. found that serum leptin levels in patients with DTC were higher and significantly decreased after thyroidectomy compared to prethyroidectomy levels [80]. Additionally, another study by Cheng et al. found that leptin and its receptor expression were positively associated with increased incidence and greater tumor size in patients with PTC [81].

Adiponectin acts as an insulin-sensitizing, anti-inflammatory, and anti-tumor agent, the latter by inhibiting cell proliferation and angiogenesis and increasing apoptosis. Low adiponectin levels observed in central obesity aggravate and are aggravated by insulin resistance, resulting in a vicious cycle with metabolic, inflammatory, and possibly oncogenic consequences [80,82]. Mitsiades et al. demonstrated that patients with TC had lower levels of circulating adiponectin than healthy controls. Therefore, circulating adiponectin is independently and inversely associated with the risk of TC [83].

## 5. Antidiabetic Agents and Thyroid Disorders

Multiple in vitro and in vivo studies have demonstrated that metformin can impede the growth of thyroid cells and different types of TC cells. Furthermore, patients with Τ2DM treated with metformin have a smaller volume of thyroid tissue and a lower risk of incident goiter, thyroid nodules, and TC [84,85,86]. Han et al. demonstrated that metformin may inhibit the growth and migration of TC cell lines through the mTOR pathway and the insulin/IGF-1 pathway [87]. Klubo-Gwiezdzinska et al. revealed that metformin inhibits TC growth in vivo and in vitro. In vitro data suggest that p70S6K/pS6 is a potential molecular target of metformin in DTC cells [88]. Another study confirmed the therapeutic efficacy of metformin in PTC in both vitro and in vivo [89]. However, more clinical studies are necessary to establish the clinical benefits of metformin in people with TC.

In some studies, adverse effects of antidiabetic drugs on thyroid function have also been observed. Metformin is a safe and effective oral hypoglycemic agent; however, it may affect thyroid function through complex pathways. Several studies suggest a TSH-lowering effect of metformin in individuals with T2DM and overt or SCH, independently of receiving TH replacement therapy, while it has no significant effects on serum levels of FT3 and FT4 [90,91]. Metformin does not affect TSH in euthyroid patients, besides those with TSH in the high normal reference range (>2.5 mU/L), in whom it can reduce TSH [91,92,93]. The impact of metformin on TSH values is reversible after its suspension. Furthermore, a study reported that metformin had a significant effect on thyroid morphology by reducing the size of the primary nodule by 30% to 50% in individuals with insulin resistance [94]. Metformin can have an impact on the course of TC through an antimitogenic and pro-apoptotic effect and by blocking the insulin proliferative effect in TC cells [95]. Another mechanism by which metformin inhibits the magnification of metastatic medullary thyroid carcinoma (MTC) is the modulation of the mTOR pathway [96]. In addition, metformin upregulates the antiproliferative effect of chemotherapeutic drugs [95]. In patients with T2DM and overt or SCH, TSH levels should be monitored after the initiation of metformin treatment.

Exogenous insulin administration can influence thyroid function in patients with T2DM by negatively regulating TRH and TSH secretion, increasing serum T4 levels, and decreasing T3 levels [97]. Regarding sulfonylureas (SUs), first-generation drugs were found to exert a goitrogenic effect on the thyroid gland, while some studies reported an increased incidence of hypothyroidism in patients treated with SUs compared to those receiving other agents [98,99,100]. However, second-generation SUs probably do not have a negative impact on thyroid function [101]. Thiazolidinediones enhance the risk of Graves’ orbitopathy (GO) and should be avoided in patients with active GO. Furthermore, they should be carefully administered to patients with T2DM with Graves’ disease to avoid adverse effects because the exacerbations they induce in GO are irreversible after discontinuation [98,102,103,104].

Regarding incretin mimetics, animal [105] and human observational [106] data suggest an increased risk of MTC after treatment with glucagon-like peptide-1 receptor agonists. However, a sub-analysis of the LEADER trial that evaluated the long-term effects of liraglutide in T2DM patients reported no adverse effects on the thyroid gland [107]. Regarding dipeptidyl peptidase-4 (DPP-4) inhibitors, a study by He et al. [108] revealed that saxagliptin promoted the migration and invasion of human TC cell lines K1 and TPC-1 in vitro and vivo through the overexpression of matrix metalloproteinase 2 (MMP2) and vascular endothelial growth factor (VEGF). MMP2 and VEGF production are upregulated by the activation of the NRF2/HO1 pathway [108]. A multicenter observational case–control study that included 80 patients with T2DM and Graves’ disease who were administered an oral hypoglycemic agent, including DPP-4 inhibitors, showed that there is a suggestive association between DPP-4 inhibitors and Graves’ disease exacerbation [109].

In patients with GO, large-dose corticosteroids are occasionally administered, which can exacerbate glucose intolerance or induce DM. Therefore, a careful assessment of the risk/benefit balance of their use in such cases is needed [110]. In contrast, in patients with T2DM and hypothyroidism, LT4 replacement may reduce insulin levels and improve endogenous insulin secretion; thus, a lower dose of insulin should be considered after normalization of thyroid function [111]. The summary of the effects of antidiabetic drugs on thyroid function is presented in Table 2.

## 6. Conclusions and Future Challenges

An increased prevalence of TD is observed among patients with T2DM, and a rise in the incidence of TC has been suggested among women with T2DM. The coexistence of these endocrinopathies seems to aggravate glycemic control and negatively affect thyroid function. In addition, specific diabetes drugs have positive or negative effects on TD that should be taken into account in daily practice. The landscape of T2DM management has been completely transformed in the last decade, with the introduction of new classes of drugs into the treatment algorithm, while a number of agents are currently in the pipeline [112,113,114]. Future studies should investigate the effects of these agents on thyroid function and morphology. It should be noted that TD is a general term that describes a heterogeneous group of diseases with variable clinical, pathophysiological, and morphological manifestations; therefore, it is essential to explore the impact of T2DM and pharmacotherapy on each clinical entity separately.

Furthermore, prospective studies with long follow-up periods are needed to assess the impact of simultaneously restoring thyroid function and achieving glycemic control on the future prognosis and the risk of complications from these correlated disorders. Currently, there are evidence gaps and, as a consequence, vagueness in guidelines on thyroid screening in patients with T2DM or regular monitoring of glycemic control in patients with TD. Taking into account the important clinical implications of the coexistence of T2DM and TD, proper screening and management strategies are needed for both endocrinopathies to ensure optimal patient care.

## Figures and Tables

**Table 1:** Prevalence of thyroid disorder in individuals with type 2 diabetes mellitus.

Study [Reference]	Publication Year	Country	Study Setting	Sample Size	Thyroid Disorders (%)
Celani et al. [14]	1994	Italy	Hospitalized patients	290	31.4
Perros et al. [10]	1995	Scotland	Hospital clinic	1310	13.4
Radaideh et al. [11]	2004	Jordan	Hospital clinic	1212	12.5
Akbar et al. [12]	2006	Saudi Arabia	Community study	200	16
Papazafiropoulou et al. [13]	2010	Greece	Outpatient clinic	1092	12.3

**Table 2:** Effect of antidiabetic drugs on thyroid function.

Metformin
TSH-lowering effect in individuals with T2DM with high normal TSH levels >2.5 mU/L and in individuals with overt or SCH
The TSH-lowering effect is independent of receiving THs replacement therapy
Reversible effect after discontinuation of metformin
A smaller volume of thyroid tissue and a lower risk of incident goiter, thyroid nodules, and TC are observed in patients with T2DM when treated with metformin
Metformin inhibits the growth and migration of TC cell lines
**Sulfonylureas**
Goitrogenic effect on the thyroid gland of the first-generation SUs
Higher incidence of hypothyroidism in patients treated with SUs compared to those receiving other agents
No impact of second-generation SUs on thyroid function
**Insulin**
Downregulation of TRH and TSH secretion
Increased serum T4 levels and decreased T3 levels
**Incretin mimetics**
Suggestive increased risk of MTC after treatment with GLP-1 receptor agonists
No adverse effects on the thyroid gland in patients with T2DM treated with liraglutide
**Dipeptidyl peptidase-4 inhibitors**
Promotion of the migration and invasion of human TC cells
Suggestive association with Graves’ disease exacerbation
**Thiazolidinediones**
Enhanced risk of GO
Irreversible exacerbations of GO even after discontinuation of thiazolidinediones

Abbreviations: T2DM: type 2 diabetes mellitus; TSH: thyroid-stimulating hormone; SCH: subclinical hypothyroidism; TH: thyroid hormones; TC: thyroid cancer; SUs: sulfonylureas; TRH: thyrotropin-releasing hormone; T4: thyroxine; T3: triiodothyronine; MTC: medullary thyroid carcinoma; GLP-1: glucagon-like peptide-1; GO: Graves’ orbitopathy.

## Data Availability

Data sharing is not applicable to this article.
